# Two conserved oligomer interfaces of NSP7 and NSP8 underpin the dynamic assembly of SARS-CoV-2 RdRP

**DOI:** 10.1093/nar/gkab370

**Published:** 2021-05-17

**Authors:** Mahamaya Biswal, Stephen Diggs, Duo Xu, Nelli Khudaverdyan, Jiuwei Lu, Jian Fang, Gregor Blaha, Rong Hai, Jikui Song

**Affiliations:** Department of Biochemistry, University of California-Riverside, Riverside, CA, USA; Department of Biochemistry, University of California-Riverside, Riverside, CA, USA; Department of Microbiology and Plant Pathology, University of California-Riverside, Riverside, CA, USA; Department of Biochemistry, University of California-Riverside, Riverside, CA, USA; Department of Biochemistry, University of California-Riverside, Riverside, CA, USA; Department of Biochemistry, University of California-Riverside, Riverside, CA, USA; Department of Biochemistry, University of California-Riverside, Riverside, CA, USA; Department of Microbiology and Plant Pathology, University of California-Riverside, Riverside, CA, USA; Department of Biochemistry, University of California-Riverside, Riverside, CA, USA

## Abstract

Replication of the ∼30 kb-long coronavirus genome is mediated by a complex of non-structural proteins (NSP), in which NSP7 and NSP8 play a critical role in regulating the RNA-dependent RNA polymerase (RdRP) activity of NSP12. The assembly of NSP7, NSP8 and NSP12 proteins is highly dynamic in solution, yet the underlying mechanism remains elusive. We report the crystal structure of the complex between NSP7 and NSP8 of SARS-CoV-2, revealing a 2:2 heterotetrameric form. Formation of the NSP7-NSP8 complex is mediated by two distinct oligomer interfaces, with interface I responsible for heterodimeric NSP7-NSP8 assembly, and interface II mediating the heterotetrameric interaction between the two NSP7-NSP8 dimers. Structure-guided mutagenesis, combined with biochemical and enzymatic assays, further reveals a structural coupling between the two oligomer interfaces, as well as the importance of these interfaces for the RdRP activity of the NSP7-NSP8-NSP12 complex. Finally, we identify an NSP7 mutation that differentially affects the stability of the NSP7-NSP8 and NSP7-NSP8-NSP12 complexes leading to a selective impairment of the RdRP activity. Together, this study provides deep insights into the structure and mechanism for the dynamic assembly of NSP7 and NSP8 in regulating the replication of the SARS-CoV-2 genome, with important implications for antiviral drug development.

## INTRODUCTION

Coronaviruses are positive-strand RNA viruses that belong to the family of *Coronaviridae*. Members of its beta-subtype have become a grave threat to public health, causing three major outbreaks in the past two decades: the Severe Acute Respiratory Syndrome-associated Coronavirus (SARS-CoV) in 2003, the Middle East Respiratory Syndrome-associated Coronavirus (MERS-CoV) in 2012, and currently, the Severe Acute Respiratory Syndrome-associated Coronavirus 2 (SARS-CoV-2) (1,2). Among these, SARS-CoV-2 has caused the current pandemic of coronavirus disease 2019 (COVID-19), with over 140 million confirmed infected cases and over three million deaths globally, leading to social, societal, and economic disruptions not seen in many generations. Despite recent progress in vaccine development, there is no highly effective therapeutic against SARS-CoV, MERS-CoV or SARS-CoV-2. To combat the current and future coronavirus outbreaks, novel therapeutics are desperately needed.

**Figure 1.**
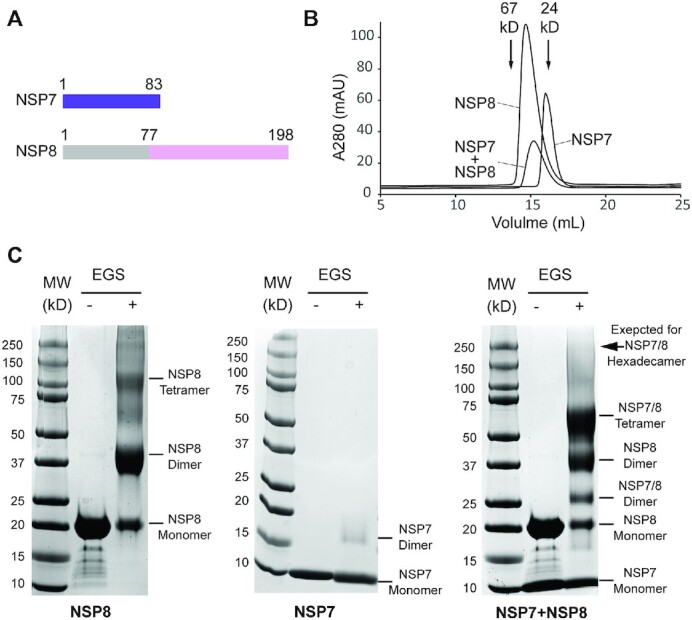
Biochemical analysis of the SARS-CoV-2 NSP7-NSP8 complex. **(A)** Domain architecture of SARS-CoV-2 NSP7 and NSP8, with the region of NSP8 missing in the crystal structure colored in grey. (**B**) Size-exclusion chromatography analysis of NSP7, NSP8 and NSP7-NSP8 complex. The elution volumes for standard proteins with known molecular weight are indicated by arrows. (**C)** SDS-PAGE images of NSP8 protein, NSP7 protein, and NSP7-NSP8 mixture, treated with or without crosslinker ethylene glycol bis(succinimidyl succinate) (EGS). The individual bands corresponding to distinct assembly states of NSP7 and NSP8 are marked. The position expected for a NSP7–NSP8 hexadecamer on the gel is indicated by arrow.

The genome of SARS-CoV-2 contains ∼30,000 nucleotides, organized into 14 open reading frames (ORFs) (3). The first ORF accounts for approximately 67% of the entire genome, encoding replicase polyproteins that are further processed by viral proteases into 15 non-structural proteins (NSPs), consisting of NSP1–NSP10 and NSP12–NSP16 (3). The central unit of the replication machinery is NSP12, which is responsible for the RNA-dependent RNA polymerase (RdRP) activity (4). In addition, NSP7 and NSP8, with molecular weights of ∼9 and ∼22 kDa, respectively (Figure 1A), serve to promote the replication processivity of NSP12 (5,6). Effective RNA synthesis is essential for the life cycle of RNA viruses, which makes the RNA replication machinery an appealing target for antiviral drug development.

Recent structural studies of the SARS-CoV NSP7-NSP8-NSP12 complex (7) and the SARS-CoV-2 NSP7–NSP8–NSP12 complex (8–12) provide mechanistic insights into the NSP12-mediated RNA elongation and the regulatory mechanism of NSP7 and NSP8. Notably, one NSP12 monomer binds to one NSP7 molecule but to two NSP8 molecules, resulting in an extended RNA binding surface spanning two turns of RNA template–RNA product duplex (10). The N-terminal helical extensions of the two NSP8 molecules form ‘sliding poles’ that interact with the downstream duplex of template and newly synthesized RNA, thus promoting the replication processivity of NSP12 (10). The crystal structure of the SARS-CoV NSP7–NSP8 complex reveals a hexadecameric architecture, with eight copies of NSP7–NSP8 heterodimer assembled into a cylindrical structure (13). However, recent studies, based on mass spectrometry and small-angle X-ray scattering (SAXS) analyses, revealed that both SARS-CoV and SARS-CoV-2 NSP7-NSP8 complexes exist as a 2:2 tetramer in solution (14,15). Along these lines, the crystal structure of the closely related feline coronavirus (FCoV) NSP7-NSP8 complex reveals a heterotrimeric complex, with two copies of NSP7 bound to the same single NSP8 molecule (16). These studies indicate a highly dynamic assembly of NSP7–NSP8 complex in solution as well as a potential function as a processivity factor for the NSP7-NSP8-NSP12 replication machinery.

To explore the molecular basis for the replication of SARS-CoV-2, we solved the crystal structure of the SARS-CoV-2 NSP7–NSP8 complex. In contrast to the hexadecameric structure observed for the SARS-CoV NSP7–NSP8 complex, the SARS-CoV-2 NSP7–NSP8 complex reveals a heterotetrameric arrangement formed by a dimer of NSP7–NSP8 dimers, with the dimerization and tetramerization of the complex mediated by two conserved, yet separate, oligomer interfaces. Importantly, mutational and biochemical analyses demonstrated that the structural integrities of the two oligomer interfaces are mutually reinforcing, resulting in a synergistic coupling between the dimerization of NSP7–NSP8 and the dimerization of the two NSP7–NSP8 dimers into the tetrameric complex. Furthermore, both interfaces engage in the assembly of the NSP7–NSP8–NSP12 complex in a similar fashion as they do for the NSP7–NSP8 complex. Consistently, mutations of the key interface residues lead to impaired RNA replication activity of the RdRP machinery. Finally, introduction of the NSP7 N37V mutation that disrupts a hydrogen bond in the NSP7–NSP8–NSP12 complex, but not in the NSP7–NSP8 complex, greatly hampers RdRP activity, thereby shedding light onto the development of potential non-nucleoside inhibitors for SARS-CoV-2 RdRP. Together, this study provides critical insights into the assembly of SARS-COV-2 NSP7–NSP8 complex and of the RdRP machinery, with important implications for the development of novel therapeutic strategies against COVID-19.

## MATERIALS AND METHODS

### Cloning, expression and purification of SARS-CoV-2 proteins

The DNA fragments encoding SARS-CoV-2 NSP7, NSP8, and NSP12 were chemically synthesized by Integrated DNA Technologies, codon optimized for bacterial expression. For structural study, the genes for full length NSP7and NSP8 were inserted in tandem into a modified pRSF Duet-1 vector, in which the NSP7 gene was preceded by an N-terminal His_6_-SUMO tag and ULP1 (ubiquitin-like protease 1) cleavage site. Next, the plasmids were transformed into BL21 (DE3) RIL cell strain (Agilent Technologies). The transformed cells were first grown at 37 °C until OD_600_ reached 0.8. The temperature was then shifted to 16 °C, followed by addition of 0.1 mM isopropyl β-D-galactoside for induction. After another 18 h of cell growth, the cells were harvested and the His_6_-SUMO-tagged NSP7 was co-purified with NSP8 using a Ni-NTA affinity column. The NSP7-NSP8 complex was then treated with ULP1 protease to remove the His_6_-SUMO tag and subjected to further purification by ion-exchange chromatography on a HiTrap Q HP Sepharose column and size-exclusion chromatography on a HiLoad 16/600 Superdex 75 pg column (GE Healthcare) pre-equilibrated with 25 mM HEPES (pH 7.5), 150 mM NaCl, 5% Glycerol, and 5 mM DTT. The purified NSP7-NSP8 complex was confirmed by SDS-PAGE, concentrated to ∼10 mg/ml, and stored at −80 °C for further use. For biochemical analysis, the genes for NSP7 and NSP8 were also individually cloned into the pRSF Duet-1 vector, and the gene for NSP12 was cloned into a modified pVP13 vector (17), N-terminally fused to a His_6_-MBP tag and a TEV cleavage site. The individual WT and mutant NSP7 and NSP8 proteins were purified in the same manner as described for the NSP7-NSP8 complex. For the RdRP assay, NSP12 protein was purified sequentially through Ni-NTA chromatography, ion-exchange chromatography on a Q HP column (GE Healthcare), tag removal via TEV cleavage, and size-exclusion chromatography on a HiLoad 16/600 Superdex 200 pg column (GE Healthcare) pre-equilibrated with 25 mM HEPES (pH 7.5), 150 mM NaCl, 5% glycerol, and 5 mM DTT. For analytical gel filtration analysis of the NSP7–NSP8–NSP12 complex, His_6_-SUMO-NSP7, His_6_-SUMO-NSP8 and His_6_-MBP–NSP12 were co-expressed in BL21 (DE3) RIL cells, and co-purified using a Ni-NTA column, followed by size-exclusion chromatography on a Superdex 200 increase 10/300 gl column (GE Healthcare). The mutations of NSP7 and of NSP8 were introduced through site-directed mutagenesis and purified in the same manner as the wild-type proteins.

For enzymatic comparison of NSP12 derived from codon-optimized and non-codon optimized gene sequences, the native NSP12-encoding DNA sequence (SARS-CoV-2 isolate: Wuhan-Hu-1/2020, NC_045512) was also inserted into the in-house His_6_-MBP vector. Expression and purification of the NSP12 protein derived from the native gene sequence followed the same procedure as that for the NSP12 protein derived from the codon-optimized gene sequence, as described above.

### Crystallization and X-ray data collection

The crystallization condition for the SARS-CoV-2 NSP7–NSP8 complex was initially identified through sparse-matrix screens (Hampton Research Inc.). The crystals were thereafter reproduced by hanging drop vapor diffusion method at 4 °C by using 1 μl of 5 mg/mL SARS-CoV-2 NSP7-NSP8 complex and 1 μL of precipitant solution (0.2 M MgCl_2_, 0.1 M HEPES (pH 7.5), 25% [w/v] Polyethylene glycol 3350). SDS-PAGE analysis of the crystals indicated that the NSP8 protein is dominated by a truncated form in crystals. For crystal harvesting, crystals were soaked in well solution supplemented with 25% glycerol before flash freezing in liquid nitrogen.

The X-ray diffraction data for the SARS-CoV-2 NSP7–NSP8 complex were collected on the Beamline 5.0.2 at the Advanced Light Source, Lawrence Berkeley National Laboratory. The diffraction data were indexed, refined, and scaled using the HKL 3000 program (18). The structure was solved by molecular replacement using the partial structure of SARS-CoV NSP7–NSP8 complex (PDB: 2AHM) as a search model. The resulting electron density revealed two molecules each for NSP7 and NSP8 in the asymmetric unit cell. The structure was further improved by iterative rounds of model building and refinement using COOT (19) and PHENIX (20) software packages. The statistics for data processing and structure refinement are summarized in Supplementary Table S1.

### Analytical size-exclusion chromatography

The solution states of NSP7, NSP8 and NSP7–NSP8 complex were analyzed using size-exclusion chromatography. In essence, 100 μl of protein solution at a concentration of 0.5 mg/ml was loaded onto Superdex 200 increase 10/300 gl column (GE Healthcare) and eluted using 25 mM HEPES (pH 7.5), 100 mM NaCl, 5% glycerol and 5 mM DTT.

### Crosslinking assay

To prepare the NSP7-NSP8 mixture, NSP7 and NSP8 proteins were each diluted to 5 mg/mL in 25 mM HEPES (pH 7.5), 150 mM NaCl, 5% glycerol and 5 mM DTT, and mixed in a 1:1 molar ratio. Next, 50 mM ethylene glycol bis(succinimidyl succinate) (EGS) dissolved in DMSO was added into 15 μl of the NSP7-NSP8 mixture to reach a final concentration of 5 mM. The reaction mixtures were then incubated on ice for 2 h before being quenched by 50 mM Tris–HCl (pH 7.5). Subsequently, the samples were subjected to SDS-PAGE analysis.

### Thermal shift assay

Thermal shift assay for NSP7 WT and mutants were conducted using a BioRad CFX Connect Real-Time PCR detection system. For each measurement, 20 μl of sample mixture contains 5.5 μM WT or mutant NSP7 dissolved in buffer containing 20 mM HEPES (pH 7.5), 10% glycerol, 150 mM NaCl, and 1× GloMelt Dye. The sample plates were heated from 25 to 95°C with heating increments of 0.5°C. Fluorescence intensity was recorded within the excitation/emission ranges of 470/510 nm. Each sample was prepared in triplicate for the measurement.

### RdRP polymerase assay

A minimal hairpin RNA substrate was used as previously reported (10). In essence, the RNA (/56-FAM/rUrUrUrUrCrArUrGrCrUrArCrGrCrGrUrArGrUrUrUrUrCrUrArCrGrCrG) was purchased from Integrated DNA Technologies. The RNA was annealed by heating the solution to 75°C and gradually cooling to 4°C in the buffer containing 10 mM HEPES (pH 7.5) and 50 mM NaCl. The polymerase assay mixture contained 5 μM RNA dissolved in 20 mM HEPES (pH 7.5), 100 mM NaCl, 5% (v/v) glycerol, 10 mM MgCl_2_ and 5 mM β-mercaptoethanol, in the presence of the indicated NSP12 (5 μM), NSP8 (15 μM) and/or NSP7 (15 μM) proteins. The reaction was initiated by addition of NTPs (150 μM UTP, GTP, and CTP and 300 μM ATP), followed by incubation at 37°C for 20, 40 and 60 min. 2× loading dye (7 M urea, 50 mM EDTA pH 8.0, 89 mM Tris-base and 28 mM Taurine) were added to stop the reaction. The reaction samples were then separated on 7 M urea, 20% acrylamide gels (8 cm × 8 cm × 1 mm) in 45 mM Tris-base, 14 mM Taurine, and 0.3 mM EDTA. 6-FAM-labeled RNA products were visualized by ChemiDoc Imager (Bio-Rad Laboratories, Inc.).

## RESULTS

### Biochemical characterization of the SARS-CoV-2 NSP7–NSP8 assembly

To examine the assembly state of NSP7 and NSP8 proteins in solution, we performed size-exclusion chromatography with NSP7, NSP8, or a mixture of NSP7–NSP8 (1:1 molar ratio). Notably, the NSP7 protein eluted at a volume close to what is expected for its monomeric form, whereas NSP8 eluted at a volume corresponding to its dimeric form (Figure 1B), in line with previous reports that NSP7 and NSP8 proteins individually exist as a monomer and a dimer in solution, respectively (16,21). On the other hand, the NSP7-NSP8 mixture eluted at a volume corresponding to what is expected for a 2:2 tetrameric form (∼62 kDa) (Figure 1B), far lower than what is expected for a hexadecameric NSP7-NSP8 complex (∼240 kDa). The caveat of protein size estimation by size-exclusion chromatography is that the elution volume of a protein can also be affected by its shape. Therefore, we also performed *in vitro* cross-linking assays on NSP7 and NSP8 using ethylene glycol bis(succinimidyl succinate) (EGS) to evaluate the assembly states of NSP7 and NSP8. SDS-PAGE analysis of EGS-treated NSP7 and NSP8 products revealed dominant monomeric and dimeric forms, respectively (Figure 1C, left and middle). Under the same reaction condition, crosslinking of the NSP7-NSP8 complex resulted in a strong SDS-PAGE band corresponding to the NSP7-NSP8 heterotetramer, but no appreciable fraction for the hexadecameric form of NSP7-NSP8 (Figure 1C, right). These observations are consistent with the recent mass spectrometry-based observation that NSP7-NSP8 is dominantly present as a tetrameric form in solution (15), suggesting that the SARS-CoV-2 NSP7–NSP8 complex exists as a 2:2 heterotetramer in solution.

### Crystal structure of the SARS-CoV-2 NSP7–NSP8 complex

**Figure 2.**
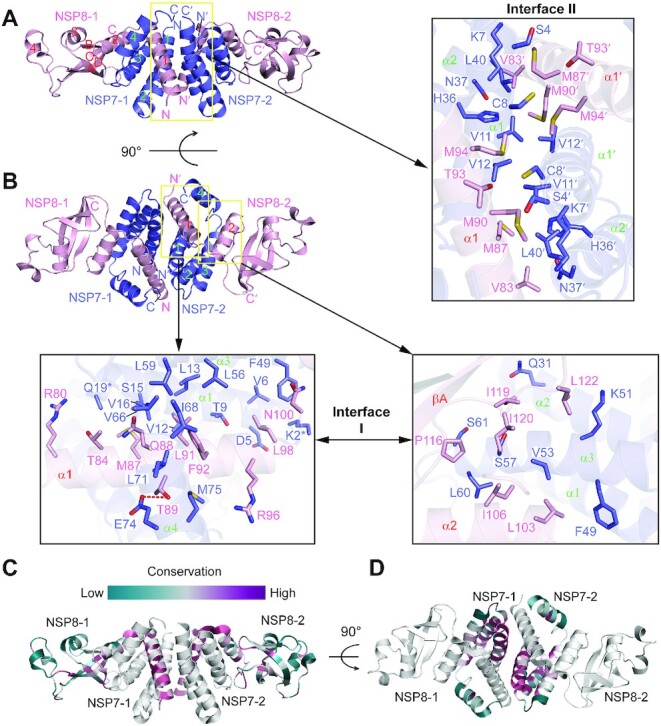
Crystal structure of the SARS-CoV-2 NSP7-NSP8 complex. (**A**, **B**) Orthogonal views of the SARS-CoV-2 NSP7-NSP8 complex, with the intermolecular interactions at the two oligomer interfaces shown in expanded views. The interacting residues are shown in stick representation. The hydrogen bond is shown as a red dashed line. The side chains of NSP7 K2 and Q19, which are untraceable in the crystal structure, are marked by asterisks. (**C**, **D**) Color-coded sequence conservation of SARS-CoV-2 NSP8 (C) and NSP7 (D), analyzed using the ConSurf server (https://consurf.tau.ac.il/) (33).

Next, we crystalized the NSP7–NSP8 complex and solved the crystal structure at 2.7 Å resolution (Supplementary Table S1). The crystal structure of the NSP7-NSP8 complex belongs to the space group *P*2_1_, with each asymmetric unit containing two NSP7 and NSP8 molecules (Figure 2A, B). Despite the fact that full-length NSP7 and NSP8 proteins were prepared for crystallization, we were only able to trace the electron density for the C-terminal domain of NSP8 spanning from residues E77 to S193, with the N-terminal portion most likely proteolytically cleaved during crystallization. On the other hand, we were able to trace the entire NSP7 molecule except for residues L83-Q84 at its C-terminus.

Analysis of the structure of the NSP7–NSP8 complex reveals a 2:2 NSP7–NSP8 tetrameric complex, formed by two closely-packed NSP7–NSP8 dimers (Figure 2A, B). As previously observed for the NSP7–NSP8–NSP12 complex (7,9–13), NSP8 is comprised of an N-terminal domain, albeit with only two α-helices traceable here, followed by a C-terminal domain formed by a four-stranded antiparallel β-sheet packed against three intervening helices (Figure 2A). NSP7 is comprised of four α-helices, with the last one moving apart from the first three to cradle the two N-terminal helices of NSP8, resulting in a mixed six-helix bundle (Figure 2B). Furthermore, helix α1 of NSP8 and helices α1–α2 of NSP7 pack against the counterparts of the other NSP7–NSP8 complex to form the tetrameric structure (Figure 2A, B). Analysis of the electrostatic surface of the NSP7–NSP8 complex failed to identify significant basic patches for potential RNA binding sites, in line with the observation that the N-terminal helices, but not the C-terminal domain, of NSP8 is responsible for RNA binding during replication or transcription of the viral genome (8,10,22).

Structural comparison of the NSP8-bound NSP7 with the previously reported solution structure of SARS-CoV NSP7 (PDB 2KYS) (21) revealed that the C-terminal half of α1, along with α2 and α3, is well aligned between the two structures, with a root-mean-square deviation (RMSD) of 2.2 Å over 51 aligned Cα atoms (Supplementary Figure S1A). The most pronounced structural deviation lies in α4, which is packed against α1–α3 in free state but breaks away in the NSP8-bound form (Supplementary Figure S1A). To test how this conformational transition of NSP7 affects its interaction with NSP8, we introduced an alanine mutation to NSP7 L71, located at the interface between α1 and α4 in the NSP7–NSP8 complex, and performed thermal shift assay. In comparison with WT NSP7, L71A-mutated NSP7 (NSP7^L71A^) shows a reduction of thermal stability by ∼9°C (Supplementary Figure S1B) but slightly increased oligomerization with NSP8 (Supplementary Figure S1C), in line with an effect of the dynamic conformational transition of NSP7 α4 on the complex formation of NSP7-NSP8.

### Structural basis for the NSP7-NSP8 interaction

Formation of the NSP7-NSP8 tetramer is mediated by two separate interfaces, with one mediating the NSP7–NSP8 dimerization (denoted as interface I in the expanded views in Figure 2B) and the other mediating the tetramerization (denoted as interface II in the expanded view in Figure 2A, herein). Close inspection of the two interfaces revealed that both the dimeric and heterotetrameric association between NSP7 and NSP8 is dominated by non-polar contacts, involving all the four helices of NSP7 and helices α1 and α2 of NSP8 (Figure 2A, B).

At interface I, residues (R80, T84, M87, Q88, T89, L91, F92, R96, L98 and N100) from helix α1 of NSP8 are clustered with residues (K2, D5, V6, T9, L13, S15, V16 and Q19) from helix α1 of NSP7 on one side and with residues (V66, I68, L71, E74 and M75) from helix α2 of NSP7 on the other side (expanded view in Figure 2B, bottom left). In addition, residues (Q31, F49, K51, V53, S57, L60 and S61) from helices α2 and α3 of NSP7 form another hydrophobic cluster with residues (L103, I106, P116, I119, I120, and L122) from helix α2 and its subsequent linker of NSP8 (expanded view in Figure 2B, bottom right). The formation of NSP7-NSP8 dimer results in a buried surface area of ∼1445 Å^2^.

Formation of interface II is mediated by helices α1 and α2 of NSP7 from one NSP7–NSP8 dimer and helix α1 of NSP8 from the other NSP7–NSP8 dimer, which are orthogonally aligned to each other to create complimentary surfaces for side-chain interactions (Figure 2A). Reciprocally, residues (S4, K7, C8, V11, V12, H36, N37 and L40) from NSP7 of one NSP7-NSP8 dimer make van der Waals contacts with residues (V83, M87, M90, T93 and M94) from NSP8 of the other NSP7-NSP8 dimer, resulting in a buried surface area of ∼773 Å^2^ (expanded view in Figure 2A).

Structure-based sequence analysis of the NSP7 and NSP8 proteins among members of the coronavirus family revealed that SARS-CoV-2 NSP7 and NSP8 are closely related to their counterparts in SARS-CoV, with 99% and 98% sequence identity, respectively, whereas the more distant FCoV NSP7 and NSP8 have only 42% and 41% sequence identity, respectively (Supplementary Figure S2A, B). The residues located on the two oligomer interfaces of NSP7 and NSP8 fall into highly conserved sites (Figure 2C, D), suggesting a conserved interaction mechanism for the NSP7–NSP8 complex formation across all coronaviruses.

### Coupling between heterodimerization and heterotetramerization of NSP7–NSP8

**Figure 3.**
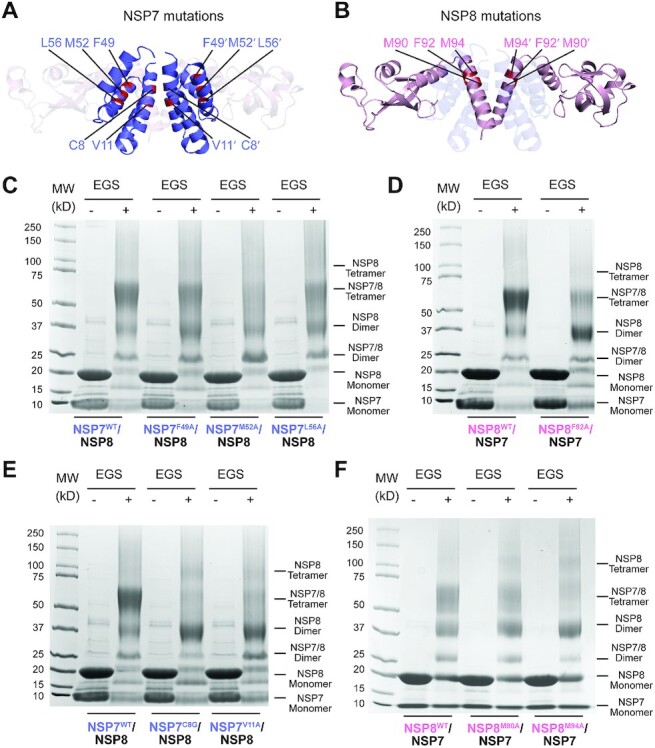
Mutational analysis of the SARS-CoV-2 NSP7–NSP8 interaction. (**A**) Residues mutated in NSP7. (**B**) Residues mutated in NSP8. (**C**, **D**) SDS-PAGE images of the NSP7-NSP8 complex, WT or mutant on the interface I of NSP7 (C) or NSP8 (D), in the presence and absence of EGS crosslinker. (**E**, **F**) SDS-PAGE images of the NSP7–NSP8 complex, WT or mutant on the interface II of NSP7 (E) or NSP8 (F), in the presence and absence of EGS crosslinker.

To further understand the structural basis for the NSP7–NSP8 assembly, we selected a number of residues from both oligomer interfaces of NSP7 and NSP8 for mutagenesis (Figure 3A, B), and evaluated their impact on the assembly of the NSP7–NSP8 complex via crosslinking assay. In this assay, we mainly evaluated the formation of NSP8 dimer, which is the dominant form of free NSP8 (Figure 1C), as opposed to NSP7–NSP8 complex formation, given that the NPS7–NSP8 complex is spread over the heterodimeric and heterotetrameric forms of the complex. The relative population of the oligomeric states of the NSP7–NSP8 complex is likely influenced by multiple structural and dynamic factors.

Inspection of the crosslinking products of the NSP7–NSP8 mixtures revealed that most of the mutations on the heterodimeric interface I of NSP7 (NSP7^F49A^ and NSP7^L56A^ in Figure 3C) and of NSP8 (NSP8^F92A^ in Figure 3D) lead to an increased NSP8 dimerization, accompanied by reduction of the heterotetramerization of NSP7–NSP8, suggesting the impairment of NSP7–NSP8 association by these mutations. Furthermore, whereas the NSP7 M52A mutation (NSP7^M52A^) does not appreciably affect the population of NSP8 dimer, it leads to a substantial heterotetramer-to-heterodimer shift of NSP7-NSP8 (Figure 3C), supporting a notion that structural integrity of interface I also affect the heterotetrameric assembly of the NSP7–NSP8 complex.

Compared with the interface I mutations of NSP7, the two interface II mutations of NSP7, C8G and V11A, lead to an even more pronounced increase of NSP8 homodimer at the expense of NSP7-NSP8 heterotetramer (Figure 3E). Note that the homotetramer band of NSP8 also appears more visible for these mutants, further supporting the notion that these interface I mutations severely disrupt the NSP7–NSP8 heterotetramer. Likewise, we observed that the NSP8 interface II mutations, M90A (NSP8^M90A^) and M94A (NSP8^M94A^), lead to an increased NSP8 dimer formation at the expense of the NSP7–NSP8 heterotetramer to an extent that is comparable or even more severe than the interface I mutation NSP8^F92A^ (compare Figure 3F with 3C). Together, these observations suggest that interface II not only serves to maintain the heterotetrameric assembly of NSP7–NSP8, but also helps to stabilize the heterodimeric assembly of NSP7–NSP8, thereby uncovering a synergistic coupling between the heterodimerization and heterotetramerization of the NSP7–NSP8 complex. Further size-exclusion chromatography analyses of the NSP7–NSP8 mixtures revealed that mutations on the interface II of NSP7 lead to even more severe loss of the tetrameric NSP7–NSP8 fraction than mutations on interface I (Supplementary Figure S3), which reinforces the notion of a structural coupling between the two oligomer interfaces of NSP7 and NSP8.

### Structural comparison of the coronavirus NSP7-NSP8 complexes

Crystal or cryo-electron microscopic (cryo-EM) structures have been reported for coronavirus NSP7–NSP8 complexes in a variety of assembly forms, including the SARS-CoV NSP7–NSP8 (13) and NSP7-NSP8-NSP12 complexes (7), the SARS-CoV-2 NSP7–NSP8–NSP12 complexes in the absence or presence of RNA substrates (10–12), as well as the NSP7–NSP8 complex from FCoV (16). These structures show diverse arrangements of the NSP7 and NSP8 proteins, including the hexadecameric arrangement of the SARS-CoV NSP7–NSP8 complex in which both NSP7 and NSP8 carry an N-terminal non-native GPLGS tag (Supplementary Figure S4A) (13), 1:2:1 heterotetrameric arrangement of the NSP7–NSP8–NSP12 complexes in which each NSP12 molecule is associated with one NSP8 monomer and one NSP7–NSP8 heterodimer (10–12), and the 1:2 heterotrimeric arrangement of the FCoV NSP7–NSP8 complex (16). Among these, the central channel of the hexadecameric complex of SARS-CoV NSP7–NSP8 (Supplementary Figure S4A) has been proposed to serve as an RNA-binding site (13), which might mediate the potential primase activity of this complex (16,23,24). However, this observation was later challenged by the fact that a SARS-CoV NSP7–NSP8 fusion protein (N7L8) had no detectable *de novo* RNA synthesis activity (6) and the biochemical evidence indicating that SARS-CoV NSP7–NSP8 exists as a tetramer in solution (14,15).

**Figure 4.**
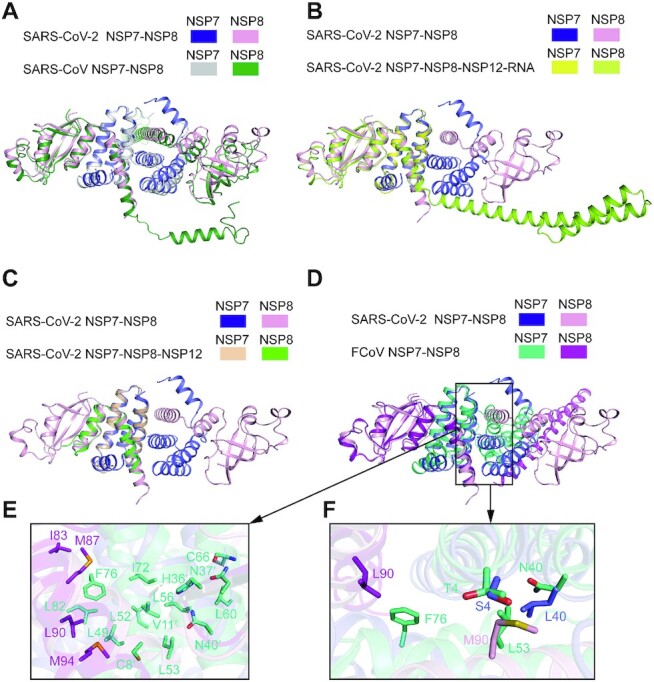
Structural analysis of the dynamic assembly of the NSP7-NSP8 complex. (**A**) Structural overlay of the SARS-CoV-2 NSP7-NSP8 tetrameric complex with the tetramer I of the SARS-CoV NSP7-NSP8 tetrameric complex (PDB 2AHM). (**B**) Structural overlay of the SARS-CoV-2 NSP7–NSP8 tetrameric complex with the SARS-CoV-2 NSP7–NSP8 dimer in the RNA-bound state of SARS-CoV-2 RdRP (PDB 6YYT). For clarity, NSP12 and the RNA molecule are omitted. (**C**) Structural overlay of the SARS-CoV-2 NSP7-NSP8 tetrameric complex with the SARS-CoV-2 NSP7–NSP8 dimer in the RNA-free state of SARS-CoV-2 RdRP (PDB 6M71). (**D**) Structural overlay of the SARS-CoV-2 NSP7–NSP8 tetrameric complex with the FCoV NSP7–NSP8 dimer in the RNA-free state of FCoV RdRP (PDB 3UB0). (**E**) Close-up view of the intermolecular interactions at the interface II of the FCoV NSP7-NSP8 complex. (**F**) Expanded view of the interface II showing NSP7 and NSP8 residues that are divergent between SARS-COV-2 and FCoV in stick representation.

Further analysis of the hexadecameric form of the SARS-CoV NSP7-NSP8 complex (PDB 2AHM) revealed that it harbors three alternative repeating units, with each adopting the form of an NSP7–NSP8 heterotetramer (Supplementary Figure S4B–D, denoted as tetramers I, II and III, respectively). Among these, formation of SARS-CoV NSP7–NSP8 tetramer I is mediated by the C-terminal domains of NSP7 and NSP8 (Supplementary Figure S4B), as is observed here for the SARS-CoV-2 NSP7–NSP8 complex (Figure 2A, B). In contrast, the formation of tetramers II and III in the SARS-CoV hexadecameric NSP7–NSP8 complex is mediated by the N-terminal helices of NSP8 proteins (Supplementary Figure S4C, D). To test the role of the N-terminal domain of NSP8 in the NSP7-NSP8 assembly, we performed size-exclusion chromatography analysis of SARS-CoV-2 NSP7 mixed with the N-terminally truncated SARS-CoV-2 NSP8 (73ΔNSP8), which showed that NSP7 and 73ΔNSP8 remain co-migrating at an elution volume corresponding to their heterotetrameric form (Supplementary Figure S4E). Consistently, crosslinking analysis of the NSP7–73ΔNSP8 mixture confirmed the predominance of the heterotetrameric form in solution (Supplementary Figure S4F). These data suggest that in solution the complex of SARS-CoV-2 NSP7–NSP8 is mainly mediated by the C-terminal domains of NSP8, rather than the long helical domain at the N-terminus. Along these lines, structural superposition of the SARS-CoV-2 NSP7–NSP8 complex with that of SARS-CoV (PDB 2AHM) shows that the SARS-CoV-2 NSP7–NSP8 tetramer is well aligned with tetramer I of the SARS-CoV NSP7–NSP8 complex, resulting in an RMSD of 0.64 Å over 458 aligned Cα atoms (Figure 4A), suggesting that the interactions mediating the hetrotetrameric assembly of NSP7-NSP8 are shared by SARS-CoV-2 and SARS-CoV.

### Oligomer interfaces I and II underpin various assembly states of NSP7, NSP8 and NSP12

Next, we asked how the oligomer interfaces of NSP7 and NSP8 undergo the transition from the heterotetrameric NSP7–NSP8 complex to the 1:2:1 heterotetrameric NSP7–NSP8-NSP12 RdRP complex. Structural superposition of the SARS-CoV-2 NSP7–NSP8 heterotetramer with the free or the RNA-bound SARS-CoV-2 RdRP reveals that the interface I-mediated NSP7–NSP8 heterodimer is preserved in the full RdRP complex (Figure 4B, C and Supplementary Figure S5A–C). Intriguingly, some of the residues of the interface II of the NSP7–NSP8 tetrameric complex (i.e. NSP7 S4, C8, V11, V12, N37 and L40 and NSP8 T84, M87, M90 and M94) engage in intermolecular contacts with NSP12 in a fashion similar to that seen in the NSP7–NSP8 complex, which is dominated by surface complementarity and hydrophobic contacts (Figure 2A and Supplementary Figure S5C). Nevertheless, distinct interaction modalities are observed for the remaining residues of interface II in the RdRP complex (Figure 2A and Supplementary Figure S5C). For instance, the side chain of NSP7 N37 donates a hydrogen bond to the backbone carbonyl group of NSP12 A443 in the RdRP complex (Supplementary Figure S5C) but interacts with NSP8 V83 side chain through a van der Waals contact in the NSP7-NSP8 heterotetrameric complex (Figure 2A). On the other side of the RdRP complex, association of the NSP8 monomer with NSP12 involves both interface I and II of NSP8 (Supplementary Figure S5D), in addition to the β-pairing mediated by the C-terminal domain of NSP8 and the polymerase domain of NSP12 (Supplementary Figure S5E). These observations suggest that interface I and II of NSP7 and NSP8 mediate the assembly of both the NSP7-NSP8 and the NSP7-NSP8-NSP12 complexes.

We further compared the structure of the SARS-CoV-2 NSP7-NSP8 heterotetramer with that of the FCoV 2:1 NSP7-NSP8 heterotrimer (Figure 4D). Despite the different stoichiometry of NSP7 and NSP8, the two complexes show high conservation for the interface I and the resulting heterodimeric structure of NSP7-NSP8 (Figure 4D). In fact, the interface II of SARS-CoV-2 NSP7–NSP8 heterotetramer also resembles the heterotrimeric interface of the FCoV complex, but with subtle differences (Figure 4E, F). For instance, NSP7 S4 and L40 interact with NSP8 M90 in the SARS-CoV-2 NSP7-NSP8 heterotetramer. In contrast, the corresponding residues in FCoV NSP7-NSP8 heterotrimer, NSP7 T4 and N40 and NSP8 L90, interact with a different set of residues (i.e. NSP7 L53 and F76) (Figure 4F). These sequence divergences may explain why the SARS-CoV-2 NSP7–NSP8 is dominated by a heterotetrameric arrangement in solution, while the FCoV NSP7–NSP8 adopts a heterotrimeric arrangement.

### Role of the NSP7-NSP8 interface residues in the activity of SARS-CoV-2 RdRP

**Figure 5.**
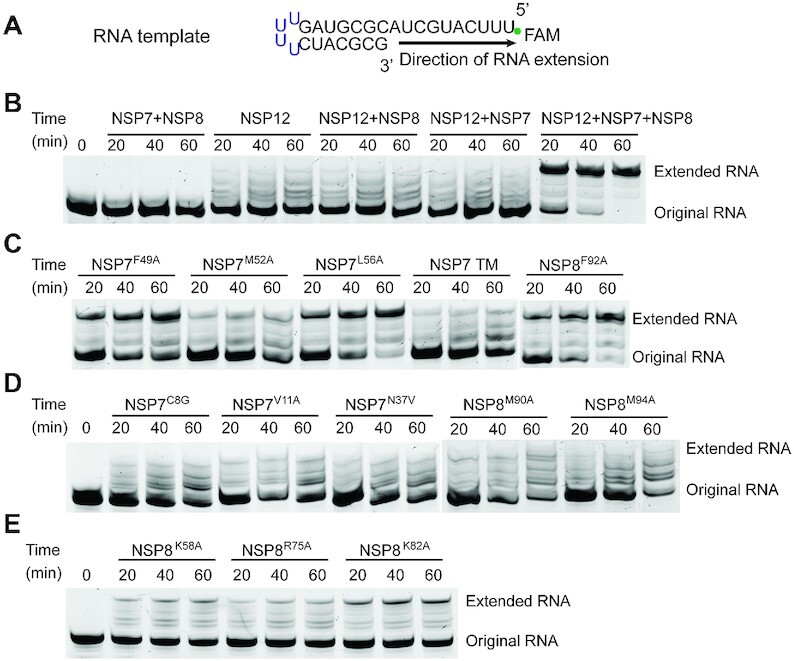
RdRP assay using a hairpin RNA substrate. (**A**) Schematic structure of the hairpin RNA. (**B**) Time-dependent incubation of NSP7, NSP8 and/or NSP12 with RNA template. (**C**) Time-dependent incubation of NSP7, NSP8 and NSP12 with RNA template, with NSP7 or NSP8 mutated at interface I. TM refers to NSP7 F49A/M52A/L56A triple mutation. (**D**) Time-dependent incubation of NSP7, NSP8 and NSP12 with RNA template, with NSP7 or NSP8 mutated at interface II. (**E**) Time-dependent incubation of NSP7, NSP8 and NSP12 with RNA template, with NSP8 mutated at the N-terminal domain.

To understand how the dynamic NSP7–NSP8-NSP12 assembly affects the RNA replication activity of SARS-CoV-2 RdRP, we next performed the primer-dependent RNA replication assay using a 5'-FAM fluorescently labeled, single-stranded RNA substrate that was recently developed (10). This 29-nt RNA folds into a hairpin structure, containing a 5 base-pair (bp) stem and an 11-nucleotide 5' overhang, which serve as the template and as the primer for efficient detection of nucleotide extension (Figure 5A).

First, incubation of WT SARS-CoV-2 NSP7–NSP8–NSP12 with the RNA substrate leads to a time-dependent increase of the extended RNA product (Figure 5B), confirming the primer-dependent replication activity of the recombinant RdRP complex. In contrast, NSP12 alone or any pairwise combination of NSP7, NSP8 and NSP12 fails to generate an appreciable level of RNA product (Figure 5B), consistent with previous observations that the co-presence of NSP7 and NSP8 greatly boosts the RNA replication efficiency of NSP12-mediated RNA replication (6,10). Second, introduction of the mutations on the interface I of NSP7 (F49A: NSP7^F49A^, M52A: NSP7^M52A^, L56A: NSP7^L56A^ and F49A/M52A/L56A: TM) or NSP8 (F92A: NSP8^F92A^) lead to a decrease of RdRP efficiency to various extents, with the NSP7 F49A/M52A/L56A triple mutation giving rise to a stronger effect than individual mutations (Figure 5C), in line with the impairments of the RdRP assembly by these mutations. Third, introduction of the mutations on the interface II of NSP7 (C8G: NSP7^C8G^ and V11A: NSP7^V11A^) or NSP8 (M90A: NSP8^M90A^ and M94A: NSP8^M94A^) lead to an even more severe reduction of RdRP efficiency (Figure 5D). Of particular note, the NSP7^C8G^ and NSP7^V11A^ mutations, which concern the association of both the NSP7-NSP8 and NSP7-NSP8-NSP12 complexes (Figure 2A and Supplementary Figure S5C), lead to nearly completely abolished activity of the RdRP complex (Figure 5D), thereby confirming the critical role of NSP7 in the RdRP activity. Together, these data reinforce the notion that the two oligomer interfaces of NSP7 and NSP8 critically mediate the assembly and RNA replication activity of the SARS-CoV-2 RdRP.

In addition, we investigated the RdRP complex carrying mutations on the potential RNA binding sites of NSP8, including K58A (NSP8^K58A^), R75A (NSP8^R75A^) and K82A (NSP8^K82A^) located on the N-terminal domain. Structural studies of the SARS-CoV-2 NSP7–NSP8–NSP12 complexes with RNA substrate bound (6,8,10,22) revealed that these residues are positioned in close proximity to the backbone of the exiting RNA duplex (Supplementary Figure S6). Indeed, all three mutations lead to a significant reduction of the RdRP efficiency (Figure 5E), confirming the important role of these residues in regulating the RdRP activity.

### NSP7 mutation with differential effect on the NSP7-NSP8 vs NSP7-NSP8-NSP12 assembly

Finally, we seek to identify any NSP7 or NSP8 mutation that perturbs the transition between NSP7–NSP8 and NSP7–NSP8–NSP12 complexes. In light of the fact that the side chain of NSP7 N37 serves as a hydrogen bond donor in the NSP7-NSP8-NSP12 complex but not in the NSP7–NSP8 heterotetramer, we mutated this residue into valine and evaluated its effect on the two complexes. Indeed, crosslinking and size-exclusion chromatography analyses revealed that, although the NSP7^N37V^ mutation does not affect the stability of the NSP7–NSP8 heterotetramer appreciably (Supplementary Figure S7A, B), it leads to a modest, but notable, disruption of the NSP7-NSP8-NSP12 complex (compare Supplementary Figure S7C and D). Consistently, the NSP7 N37V mutation significantly compromises the replication efficiency of the NSP7–NSP8–NSP12 complex (Figure 5D). The identification of the NSP7 N37V mutation causing differential effects on various assembly states of RdRP provides a new avenue for the development of allosteric inhibitors that specifically inhibit SARS-CoV-2 RdRP activity.

It is worth noting that a recent study indicated that bacterial expression of the codon-optimized NSP12 affects its translational rate, thereby compromising its co-translational folding and consequently, the activity of the RdRP complex (25). To ensure the proper folding of the recombinant NSP12 protein used in this study, which was codon-optimized for protein expression in E. Coli, we expressed the NSP12 protein in the form of an MBP-fusion protein using a modified pVP13 vector with a T5 promoter (17). Our RdRP assays indicate that, in comparison with the NSP12 protein sample encoded by a native NSP12-coding sequence (SARS-CoV-2 isolate: Wuhan-Hu-1/2020, NC_045512), the NSP12 protein sample derived from the codon-optimized construct shows a similar activity, albeit with a slightly higher accumulation of reaction intermediates (Supplementary Figure S8A,B). This observation therefore validates the NSP12 sample used in our enzymatic assays.

## DISCUSSION

The recurrent outbreaks of viruses call for the development of highly efficient inhibitors targeting the fundamental machinery that underpins viral infections, such as RdRP (26). Uniquely among RNA viruses, NSP7, NSP8, and NSP12 proteins constitute the core components of the coronavirus RdRP machinery that mediates viral replication. Through combined structural, biochemical, and enzymatic analyses, our study uncovers the molecular basis for the dynamic assembly of the NSP7-NSP8 complex and its relationship to RdRP activity, thereby providing insights into the functional regulation of viral replication.

**Figure 6.**
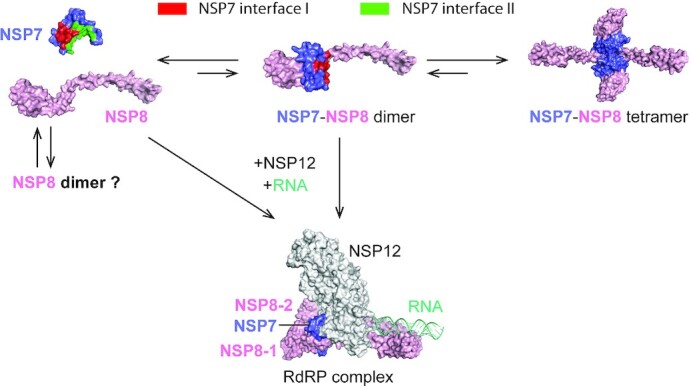
A model for the dynamic assembly of the NSP7–NSP8 complex. The mixed populations of NSP7 monomer, NSP8 monomer, NSP8 dimer, NSP7–NSP8 heterodimer, and NSP7–NSP8 heterotetramer co-exist in solution. The population of NSP7–NSP8 heterodimer is transient due to coupled intermolecular interactions between interface I and interface II. In the presence of NSP12 and RNA, NSP7–NSP8 heterodimer and NSP8 monomer associate with NSP12 to form a functional RdRP complex.

This study reveals that the SARS-CoV-2 NSP7–NSP8 complex adopts a heterotetrameric structure in solution. Formation of the NSP7–NSP8 complex involves two related, yet separate, non-polar interfaces, which mediate the heterodimeric and heterotetrameric assembly of NSP7–NSP8 in a synergistic manner (Figure 6). The heterotetrameric interface (interface II) is formed by the N-terminal helices of NSP7 and NSP8, which contribute to relatively conserved yet distinct conformations in the different NSP7-NSP8 assembly states (Figure 4 and Supplementary Figure S4A–D). The coupling between the heterodimerization and the heterotetramerization of SARS-CoV-2 NSP7–NSP8 likely arises from the fact that the two oligomer interfaces are formed by a distinct, yet overlapping set of structural elements (i.e. NSP7 α1 and NSP8 α1): Formation of the SARS-CoV-2 NSP7–NSP8 heterotetramer presumably leads to reduced conformational entropy of NSP7 α1 and NSP8 α1, which in turn stabilizes the interface I-mediated heterodimeric interactions (Figure 6). Note that all three components of the SARS-CoV-2 RdRP complex have recently been shown to possess a RNA replication-independent function (27). In this context, this coupled dimerization-tetramerization of the NSP7-NSP8 complex may not only help to shield the NSP7–NSP8 from unwanted protein interactions, but also provide a mechanism for the dynamic transition between different functional states of NSP7 and NSP8. During the assembly of the RdRP complex, the residues on the interface II of NSP7 and NSP8 interact with NSP12 in a similar manner as that in the NSP7-NSP8 heterotetramer, leading to a shift from the NSP7-NSP8 tetramer to the NSP7–NSP8–NSP12 complex (Figure 6). The residues on both interfaces are highly conserved across different coronaviruses, in line with their important roles in mediating the assembly of the RdRP and NSP7-NSP8 complexes. This study therefore reveals an unprecedented NSP7–NSP8 interaction mechanism, with important implications in the functional regulation of the RdRP complex during the replication or other stage of the viral infection.

SARS-CoV-2 has been mutating since its emergence, resulting in the appearance of several variants. These new SARS-CoV-2 variants also carry mutations in NSP7, NSP8 and NSP12, including the frequently detected NSP12 P323L, NSP7 S25L and NSP8 M129I and I156V mutations (28). Among these, NSP12 P323L has been identified as one of the hot-spot mutations associated with increased severity of COVID-19 (29,30), suggesting a link to an increased transmission capacity of the SARS-CoV-2 variants carrying this mutation. Structural analyses of the NSP7–NSP8 and NSP7–NSP8–NSP12 complexes revealed that the P323L mutation is located next to one of the NSP8–NSP12 interfaces (Supplementary Figure S9A). Replacement of NSP12 P323 with a leucine likely results in enhanced van der Waals interaction between NSP8 and NSP12. Likewise, the NSP7 S25L mutation is located near to the NSP7-NSP8 interface in the NSP7–NSP8–NSP12 complex (Supplementary Figure S9B); replacement of NSP7 S25 with a bulky phenylalanine therefore may lead to enhanced NSP7–NSP8 association within the RdRP complex. In addition, the NSP8 M129I mutation is mapped onto the interface between NSP12 and the second NSP8 molecule (Supplementary Figure S5E). However, given the fact that this interface is mainly mediated by a β-pairing between NSP8 and NSP12, the NSP8 M129I mutation may not generate any significant impact on the NSP8–NSP12 association. On the other hand, none of these frequent NSP7 or NSP8 mutations mentioned above are located at the oligomer interfaces of the NSP7–NSP8 tetramer (Supplementary Figure S9C, D), supporting the functional relevance of the NSP7–NSP8 tetramer.

Our study also demonstrates that the dynamic equilibrium between different assembly states of NSP7-NSP8 can be fine-tuned by the interface mutations. Note that the residues on the oligomer interfaces are highly conserved across coronaviruses, highlighting their functional importance. Introduction of the NSP7^N37V^ mutation affects the stabilities of the NSP7–NSP8 and NSP7–NSP8–NSP12 differently, leading to a population shift from the NSP7–NSP8–NSP12 complex toward the NSP7–NSP8 complex in solution, and consequent impairment of the RdRP activity. Conceivably, the NSP7 protein carrying the N37V-like mutations could be exogenously introduced into the infected cells to interact with NSP8 to form an NSP12 binding-defective complex, thereby interfering with the assembly of an active viral RdRP complex. In this context, exogenous NSP7 with N37V-like mutations may serve to deplete the pool of NSP8 proteins available for RdRP formation in infected cells, leading to allosteric inhibition against SARS-CoV-2. Whether this allosteric inhibition scheme can serve as a novel therapeutic strategy to complement existing nucleoside analogue-based treatment (e.g. Remdesivir) (31) awaits future investigation.

While this study was in process, two other groups reported the crystal structures of the SARS-CoV-2 NSP7-NSP8 complex (PDB 6YHU, 6M5I and 6WIQ) (32). All of these structures are consistent with our observed heterotetrameric assembly of NSP7–NSP8.

## DATA AVAILABILITY

Coordinates and structure factors for the SARS-CoV-2 NSP7–NSP8 complex have been deposited in the Protein Data Bank under accession code 7JLT.

## Supplementary Material

gkab370_Supplemental_File
