# Supplementary material for: Two conserved oligomer interfaces of NSP7 and NSP8 underpin the dynamic assembly of SARS-CoV-2 RdRP
Source: Nucleic Acids Res. 2021 May 17;49(10):5956–66. doi: 10.1093/nar/gkab370 (PMC8191759; doi:10.1093/nar/gkab370)
Supplement: gkab370_Supplemental_File [file gkab370_supplemental_file.pdf]

Supplementary Information

Two conserved oligomer interfaces of NSP7 and NSP8 underpin the dynamic assembly of SARS-CoV-2 RdRP

Mahamaya Biswal[1], Stephen Diggs[1], Duo Xu[2], Nelli Khudaverdyan[1], Jiuwei Lu[1], Jian Fang[1], Gregor Blaha[1], Rong Hai[2], Jikui Song[1]

[1]Department of Biochemistry, University of California, Riverside, California, USA

[2]Department of Microbiology and Plant Pathology, University of California, Riverside, California, USA

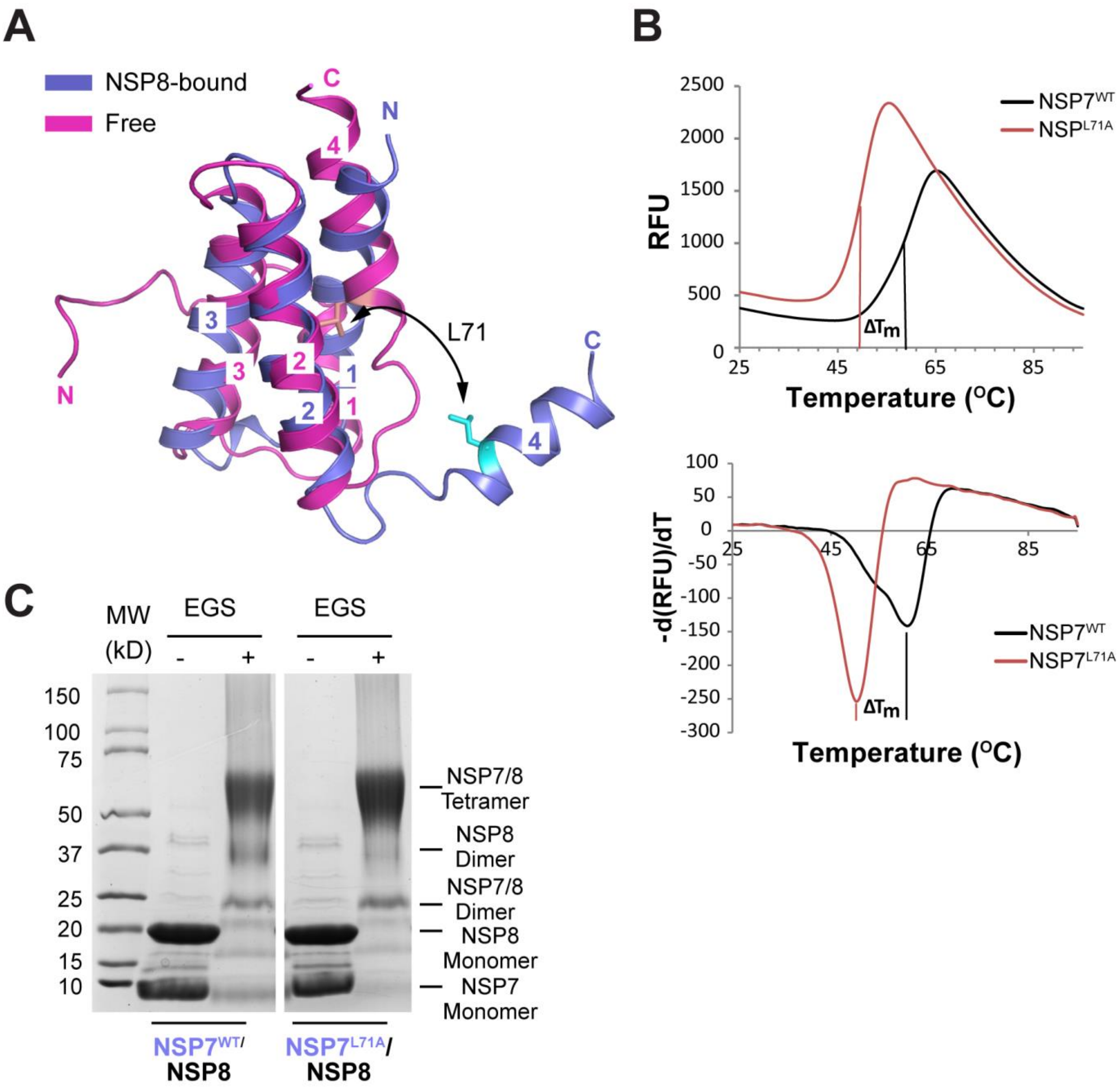

Figure S1. Structural and biochemical analysis of the conformational dynamics of NSP7. (A) Structural overlay of NSP8-bound NSP7 of SARS-CoV-2 with SARS-CoV NSP7 in free state (PDB 2KYS). **(B)** Thermal shift assay for the wild type ($NSP7^{WT}$) and L71A-mutated NSP7 ($NSP7^{L71A}$), with raw fluorescence data (top) and first derivative of the raw data (bottom) shown. The difference in melting temperature (ΔT_m) between $NSP7^{WT}$ and $NSP7^{L71A}$ is indicated. **(C)** SDS-PAGE image showing WT and NSP7 L71A-mutated SARS-CoV-2 NSP7-NSP8 mixture in the presence and absence of ethylene glycol bis(succinimidyl succinate) (EGS) crosslinker.

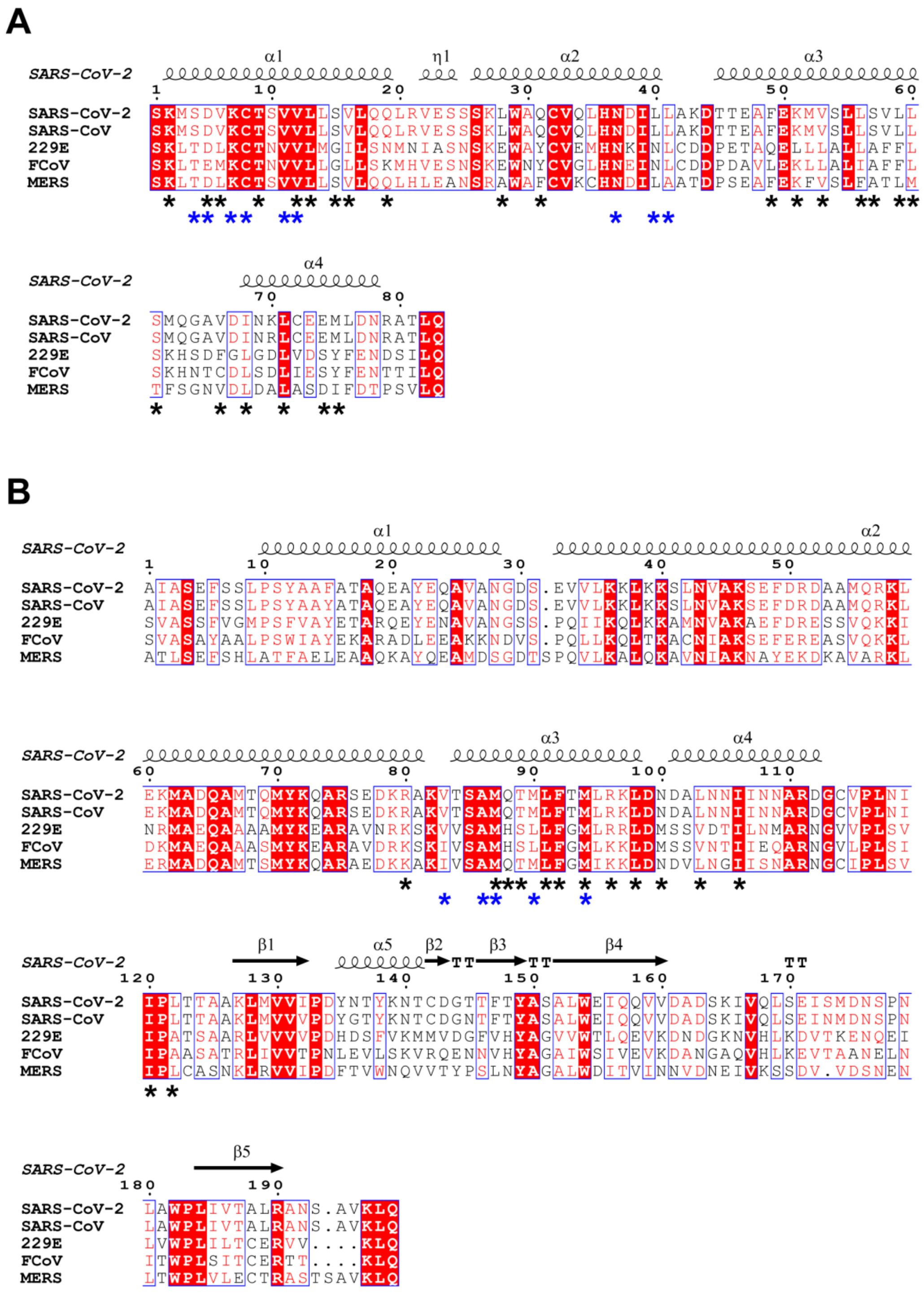

Figure S2. Sequence alignment of SARS-CoV-2 NSP7 (A) and NSP8 (B) with homologues from other coronaviruses. Identical or similar residues are boxed and colored in red. Completely conserved residues are shaded in red. The secondary structures of SARS-CoV-2 NSP7 and NSP8 are marked above the aligned sequences and residues located on interfaces I and II are marked below the aligned sequences by black and blue asterisks, respectively.

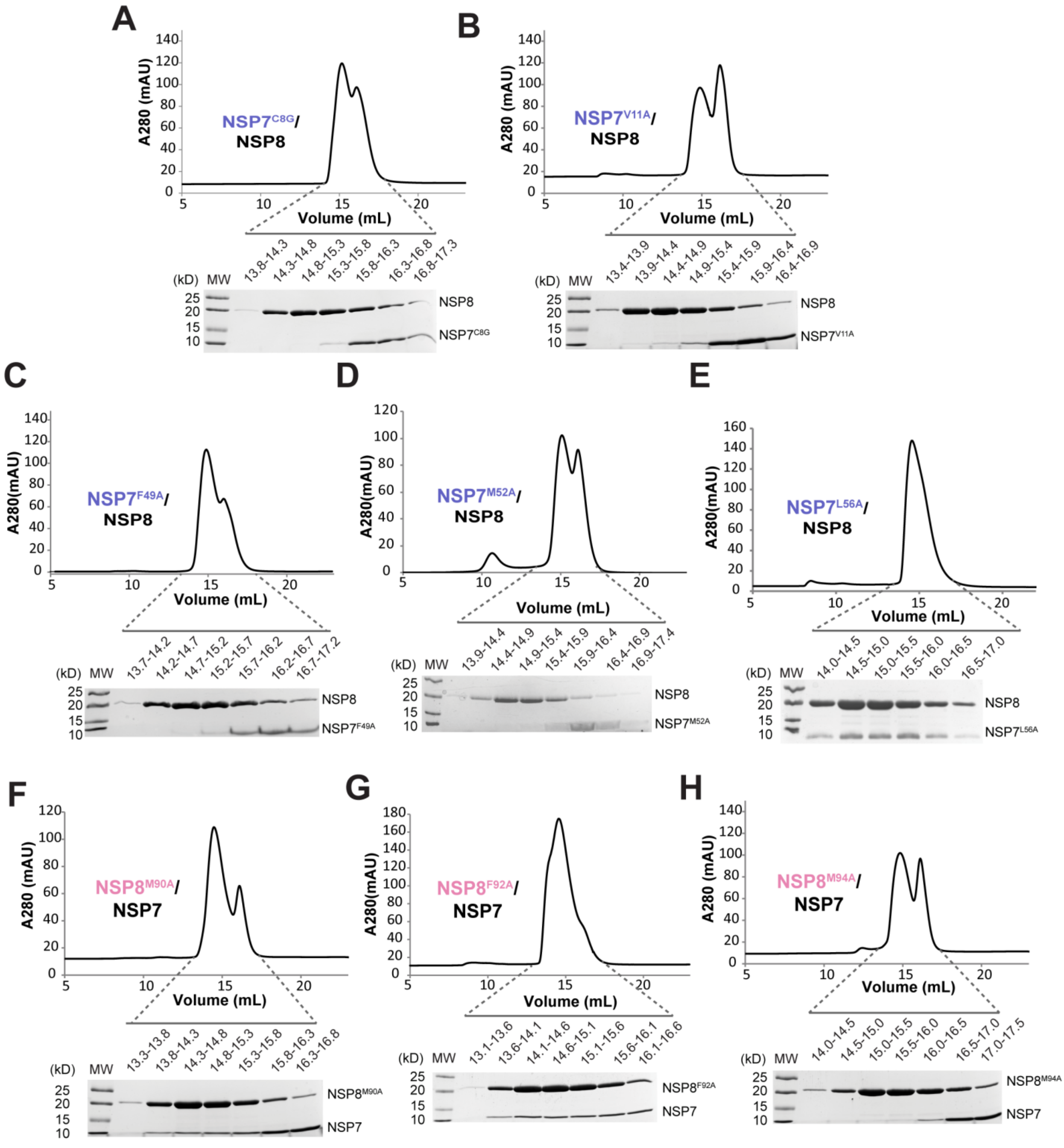

Figure S3. Size-exclusion chromatography analyses of NSP7-NSP8 mixtures. (A-E) Gel filtration profile of the NSP7-NSP8 mixture harboring NSP7^{C8G} (A), NSP7^{V11A} (B), NSP7^{F49A} (C), NSP7^{M52A} (D), or NSP7^{L56A} (E) mutations. **(F-H)** Gel filtration profile of the NSP7-NSP8 mixture harboring NSP8^{M90A} (F), NSP8^{F92A} (G), or NSP8^{M94A} (H) mutations. The SDS-PAGE images for selected fractions are shown below.

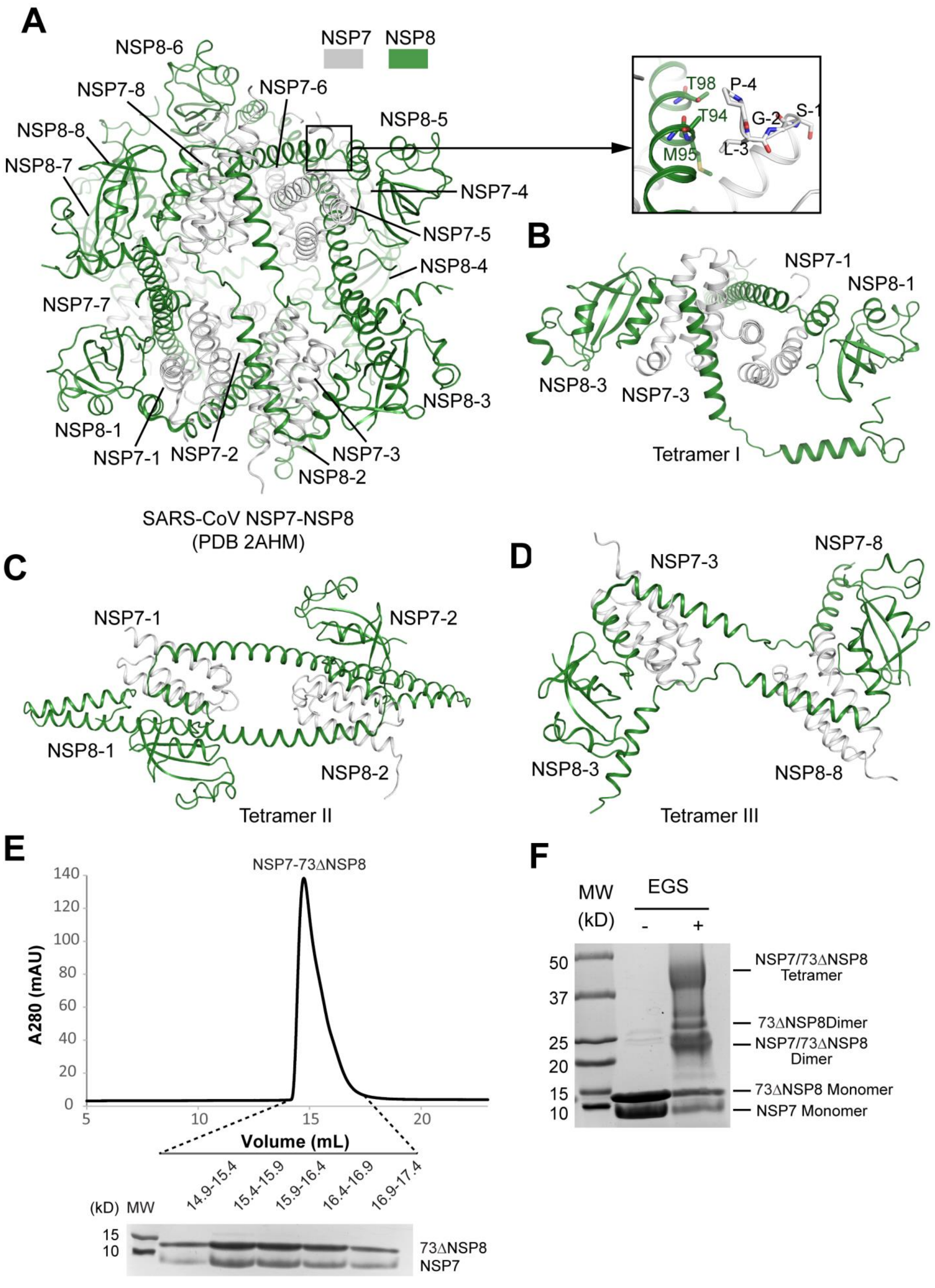
A
NSP7
NSP8
NSP8-6
NSP7-8
NSP7-6
NSP8-5
NSP8-8
NSP8-7
NSP7-4
NSP7-5
NSP8-4
NSP7-7
NSP8-3
NSP8-1
NSP7-1
NSP7-2
NSP7-3
NSP8-2
SARS-CoV NSP7-NSP8
(PDB 2AHM)
T98
P-4
T94
G-2
S-1
M95
L-3
B
NSP7-1
NSP8-1
NSP8-3
NSP7-3
Tetramer I
C
NSP7-1
NSP7-2
NSP8-1
NSP8-2
Tetramer II
D
NSP7-3
NSP7-8
NSP8-3
NSP8-8
Tetramer III
E
NSP7-73ΔNSP8
A280 (mAU)
140
120
100
80
60
40
20
0
5
10
15
20
Volume (mL)
14.9-15.4
15.4-15.9
15.9-16.4
16.4-16.9
16.9-17.4
(kD)
MW
15
10
73ΔNSP8
NSP7
F
MW
(kD)
EGS
-
+
50
37
25
20
15
10
NSP7/73ΔNSP8 Tetramer
73ΔNSP8Dimer
NSP7/73ΔNSP8 Dimer
73ΔNSP8 Monomer
NSP7 Monomer

Figure S4. Structural and biochemical analysis of the oligomeric state of NSP7-NSP8. (A) Ribbon representation of the crystal structure of the SARS-CoV NSP7-NSP8 hexadecameric complex, with individual NSP7 and NSP8 subunits labeled (NSP7-1 to NSP7-8 and NSP8-1 to NSP8-8). The N-terminal GPLGS tag of NSP8 participated in the formation of hexadecameric complex, as shown in the expanded view, which may contribute to the stability of the hexadecameric form of the complex in the crystal structure. **(B-D)** Three alternative heterotetrameric assembly of SARS-CoV NSP7-NSP8 derived from the hexadecameric structure in (A). **(E)** Size-exclusion chromatography analysis of the complex formation of NSP8 with N-terminal helix deletion (73Δ NSP8) with NSP7. Shown beneath the chromatogram is the SDS-PAGE image of the peak fractions. **(F)** SDS-PAGE image shows the NSP7-73ΔNSP8 mixture treated with EGS. The individual bands corresponding to distinct assembly states of NSP7 and NSP8 are marked.

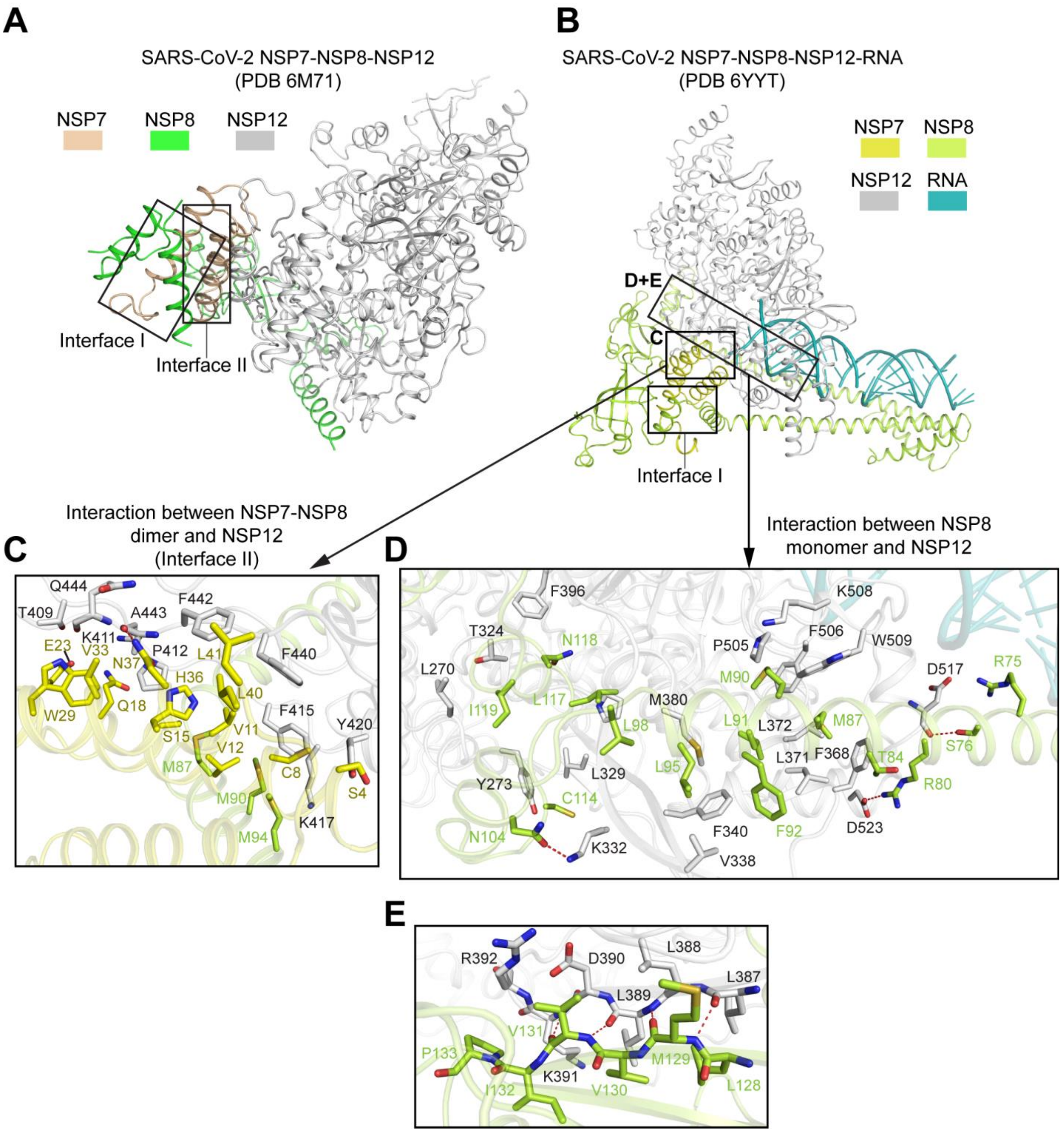

Figure S5. Structural analysis of SARS-COV-2 NSP7-NSP8 in the context of the RdRP complex. (A) Cryo-EM structure of NSP7-NSP8-NSP12 (PDB 6M71), with individual subunits color coded. **(B)** Cryo-EM structure of NSP7-NSP8-NSP12-RNA (PDB 6YYT), with individual subunits color coded. **(C)** Expanded view of (B) showing the intermolecular interactions between NSP7-NSP8 and NSP12. **(D-E)** Expanded view of (B) showing the intermolecular interactions between NSP8 monomer and NSP12 involving non-polar contacts (D) and β pairing (E).

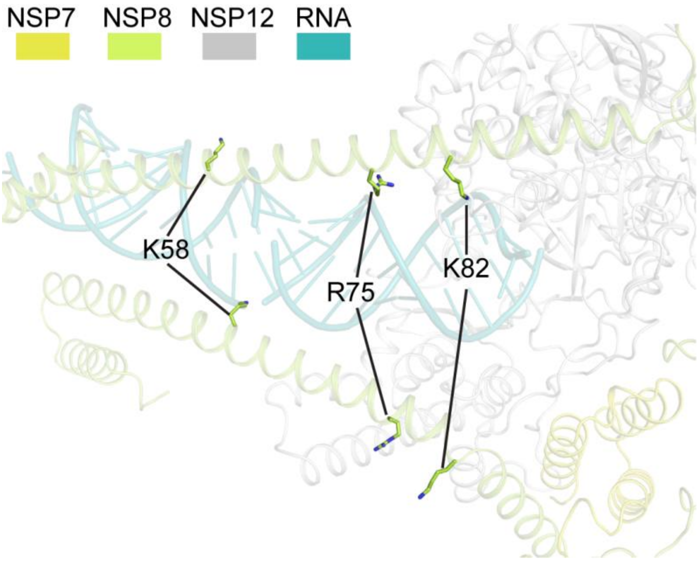

Figure S6. Selected RNA-binding sites on NSP8 in the structure of NSP7-NSP8-NSP12-RNA complex (PDB 6YYT). The side chains of NSP8 K58, R75 and K82 are shown in stick representation.

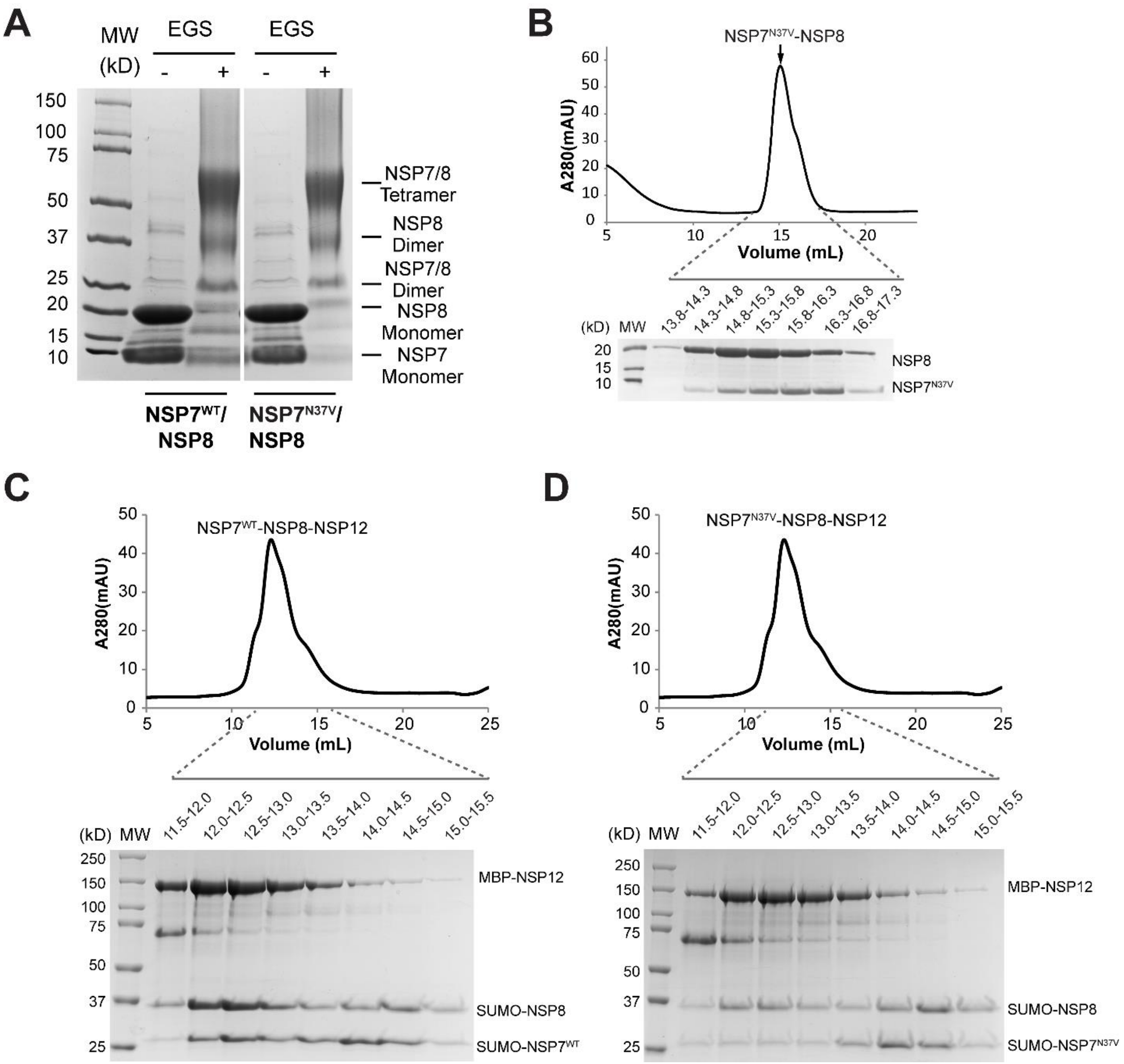

Figure S7. Biochemical analysis of NSP7 N37V mutant. (A) SDS-PAGE images of the NSP7-NSP8 mixture harboring WT or N37V mutant, in the presence and absence of EGS crosslinker. **(B)** Gel filtration profile of NSP7^{N37V}-NSP8 mixture, with the SDS-PAGE images of peak fractions shown below. **(C, D)** SDS-PAGE images showing gel filtration fractions of SUMO- NSP7^{WT} mixed with SUMO-NSP8 and MBP-NSP12 (C) and SUMO-NSP7^{N37V} mixed with SUMO-NSP8 and MBP-NSP12.

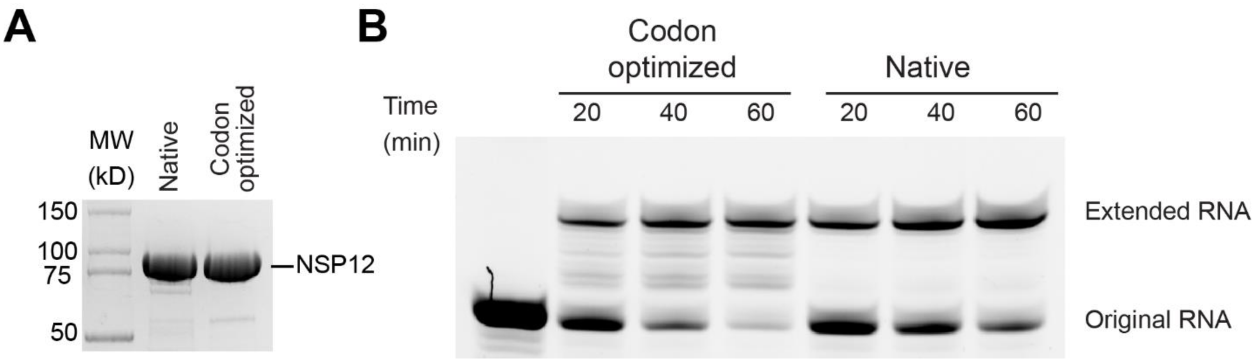

Figure S8. Enzymatic comparison of the NSP12 proteins derived from the gene sequence with different codon usage. (A) SDS-PAGE images of the NSP12 proteins derived from the gene sequences either native (Wuhan-Hu-1/2020, NC_045512) or codon-optimized for bacterial expression. **(B)** Time-dependent RdRP assay with the NSP12 protein, derived from either the native or codon-optimized gene sequence, co-incubated with NSP7 and NSP8 proteins.

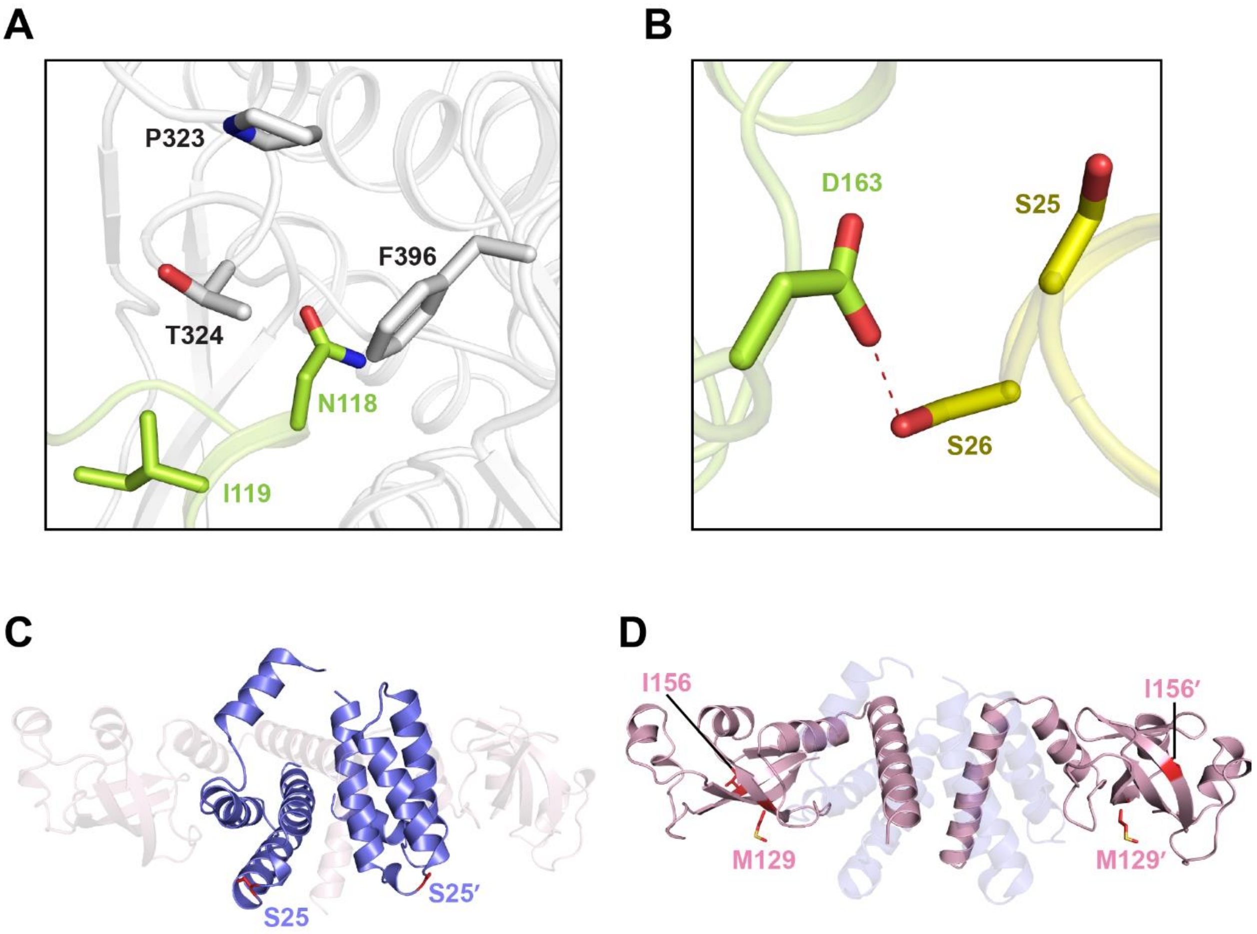

Figure S9. Structural analysis of the NSP7 and NSP8 mutation sites from new SARS-CoV-2 variants. (A) Close-up view of NSP12 P323 and its surrounding protein residues in the NSP7-NSP8-NSP12-RNA complex (PDB 6YYT). NSP12 and NSP8 are colored in grey and lime green, respectively. **(B)** Close-up view of NSP7 S25 and its surrounding protein residues in the NSP7-NSP8-NSP12 complex (PDB 6YYT). NSP7 and NSP8 are colored in yellow and lime green, respectively. Note that the neighboring S26 of NSP7 forms a side-chain hydrogen bond with NSP8 D163. **(C, D)** Mapping of NSP7 S25 (C) and NSP8 M129/I156 (D) onto the structure of the NSP7-NSP8 tetramer in this study (PDB 7JLT).

Table S1. Data collection and refinement statistics

	SARS-CoV-2 NSP7-NSP8 (PDB 7JLT)
**Data collection**	
Space group	$P\ 2_1$
Cell dimensions	
a, *b*, *c* (Å)	42.8, 110.6, 42.9
α, β, γ (°)	90, 105.7, 90
Resolution (Å)	41.3-2.7 (2.8-2.7)[a]
R_{merge}	0.129 (0.817)
$I/\sigma(I)$	8.3 (1.1)
$CC_{1/2}$	0.994 (0.628)
Completeness (%)	98.6 (93.1)
Redundancy	4.0 (3.9)
**Refinement**	
Resolution (Å)	41.3-2.7 (2.8-2.7)
No. reflections	10405 (956)
R_{work} / R_{free}	23.7/28.3 (30.4/37.5)
No. atoms	
Protein	2935
Water	50
B factors (Å^2)	
Protein	57.93
Water	50.62
R.m.s. deviations	
Bond lengths (Å)	0.008
Bond angles (°)	1.481

[a] Values in parentheses are for highest-resolution shell.
The data were collected from a single crystal.
